# Upper gastrointestinal bleed secondary to large right hepatic artery pseudoaneurysm: a case report

**DOI:** 10.1093/omcr/omae090

**Published:** 2024-08-19

**Authors:** Swotantra Gautam, Aakash Neupane, Gurdeep Singh, Mohamad Sharbatji

**Affiliations:** Department of Internal Medicine, Advent Health, 601 East Rollins Street, Orlando, Florida 32803, United States; Department of Internal Medicine, B.P. Koirala Institute of Health Sciences, Dharan-18, Sunsari 56700, Nepal; Department of Internal Medicine, Advent Health, 601 East Rollins Street, Orlando, Florida 32803, United States; Department of Internal Medicine, Advent Health, 601 East Rollins Street, Orlando, Florida 32803, United States

**Keywords:** gastroenterology, hepatic artery pseudoaneurysm, upper gastrointestinal bleed

## Abstract

Hepatic artery pseudoaneurysm (HAP) is a serious rare life-threatening complication of Gastrointestinal surgeries that is often overlooked in diagnostic evaluation due to its rarity. We present a case of 71 years female, with a surgical history of gastric sleeve surgery, Roux-en-Y gastric bypass, and cholecystectomy, presenting with features of upper GI bleeding. Multiple diagnostic modalities were used and finally Magnetic Resonance Mesenteric Angiogram was able to pinpoint the location of the GI bleed as a hepatic artery pseudoaneurysm. Primary surgical repair used to be the mainstay treatment option for managing visceral aneurysms. However, due to advances in technology, embolization as well as implantation of covered stent grafts have become the preferred treatment for such lesions.

## Introduction

An arterial pseudoaneurysm results from turbulent blood flow at any artery following puncture, forming a hematoma with a non-closing-neck beyond a specific size. It usually occurs in the splenic artery (most frequent), gastroduodenal artery, pancreatoduodenal artery, superior mesenteric artery, left gastric artery, hepatic artery, and small intrapancreatic arteries [1]. Hepatic artery pseudoaneurysm is characterized by a pulsating hematoma resulting from blood leakage through a tear or disruption in the arterial wall, with containment occurring only by the hepatic parenchyma or surrounding hematoma. The prevalence of hepatic artery aneurysms is estimated to be around 0.002%, and approximately 50% of these cases are pseudoaneurysms. Various factors such as iatrogenic and traumatic cases, arteriosclerotic disease, vasculitis, pancreatitis, cholecystitis, and gastrointestinal surgery have been associated with the development of this clinical condition [2]. Such pseudoaneurysms pose a significant risk of spontaneous rupture, leading to a potentially catastrophic vascular event in the abdomen. Reported mortality rates following the rupture of any visceral artery aneurysm range between 25% and 70% [3].

Herein, we present a 71-year-old female patient with a hepatic artery pseudoaneurysm possibly due to previous hepatobiliary system surgery who presented with upper gastrointestinal system bleeding.

## Case presentation

**Figure 1:**
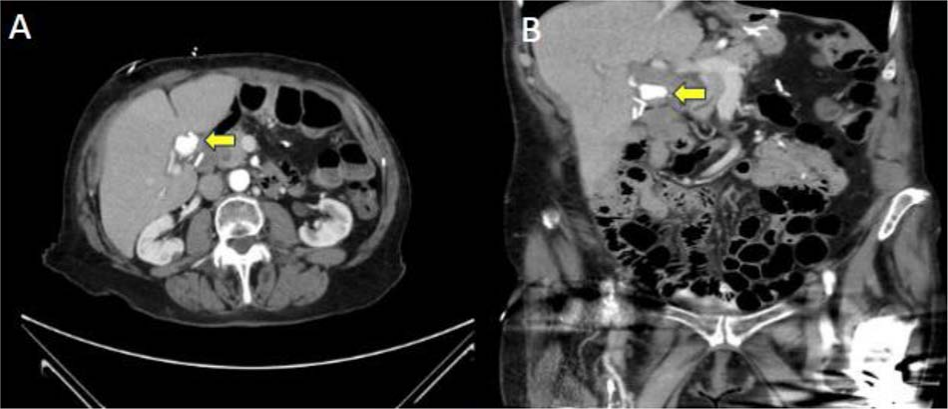
Computed tomography (CT) of the abdomen/pelvis in axial (**A**) and coronal (**B**) demonstrating a hyper-enhancing mass in the hepatic hilum (marked by arrow).

A 71-year-old female with a history of hypertension, hyperlipidemia, hypothyroidism, chronic pain, depression, and bipolar disorder, surgical history of gastric sleeve surgery, Roux-en-Y gastric bypass with several revisions, cholecystectomy, appendectomy, and splenectomy and under outpatient medications Ramipril for Hypertension, Atorvastatin, Levothyroxine, Wellbutrin, Buspar, Celexa, Aricept and Trazodone initially presented to the ED with upper Gastrointestinal bleed in the form of malena as well as chest pain and abdominal pain. She was evaluated and found to have intrahepatic ductal obstruction with cholangitis leading to septic shock. Managed in ICU with antibiotics, vasopressors, and oxygen support, her initial EUS showed no significant hepatobiliary pathology. DB-ERCP was attempted but cannulation failed. A large clean-based marginal ulcer at the gastrojejunal anastomosis, initially thought to cause melena, was treated with PPIs and Sucralfate. On repeat EUS, profuse bile drainage ruled out biliary obstruction. The patient continued antibiotics and showed hemodynamic improvement and was afebrile. CT Abdomen and Pelvis reported a round radiodensity in the hepatic hilum adjacent to surgical clips (Fig. 1). Differentials such as a stone in the cystic duct remnant, lymph node, mass, or post-surgical change such as organized hematoma or chronic fat necrosis were postulated. Chest pain was nonspecific, relieved in due course of time and no further cardiovascular evaluation was needed.

**Figure 2:**
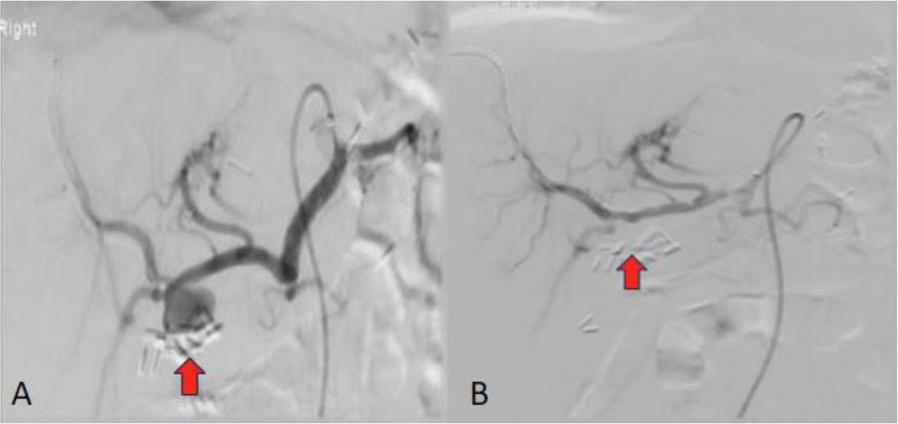
Arteriogram of the right hepatic artery demonstrating a pseudoaneurysm (marked by arrow) adjacent to surgical clips before (**A**) and after endovascular stenting (**B**).

However, the patient again reported positive melena on the morning of Day 6 of ICU. MRI Abdomen was done and it showed that the mass in the hepatic hilum represents a right hepatic artery pseudoaneurysm measuring 1.9 cm (Fig. 2A). This was the likely suspect for gastrointestinal bleeding. The patient was stable after a blood transfusion and Mesenteric Angiogram was done and a covered stent was placed in the right hepatic artery (Fig. 2B).

## Discussion

Hepatic artery pseudoaneurysm (HAP) is a serious complication of injury to the hepatic artery. It usually occurs after acute/chronic severe pancreatitis, blunt abdominal trauma, or iatrogenic causes such as liver biopsy, percutaneous transhepatic biliary drainage and surgical treatment such as laparoscopic cholecystectomy, lymph node dissection for malignancy or any hepatobiliary system surgeries. Laparoscopic cholecystectomy (LC) is most commonly associated with HAP as 7% of LC is associated with bile duct injury [4]. The mechanism by which HAP develops after surgery is postulated to be a direct mechanical vascular injury, erosion due to clip encroachment and infection associated with diathermy shortening on clips. Bile leak and secondary infection are the most important factors. Bile is cytotoxic when present in abnormally high concentrations and this leads to digestion of the hepatic arterial wall due to infectious bile from anastomotic leakage and arterial irritation due to a localized abscess leading to the formation of a Pseudoaneurysm [5]. The risk is especially high if the patient has had a bile leak after the initial surgical repair of a bile duct injury [6]. In our case, the patient had sleeve bariatric surgery in 2017, which was converted to a gastric bypass in 2018. In 2019, complications included a Roux limb perforation, splenic laceration requiring splenectomy, and gallstones necessitating a cholecystectomy; which are a risk factor in developing hepatic artery injury and subsequent hepatic artery pseudoaneurysm. HAPs can be found incidentally but often present haemobilia (90%), abdominal pain (70%), and jaundice (60%). Less than 40% present with the classic Quincke’s Triad (jaundice, biliary colic, and gastrointestinal bleeding) [7, 8]. Most patients typically present within 6 weeks of the insult, but in rare instances, presentation may be delayed by over a year [6]. In our case, the patient developed pseudoaneurysm five years later after major GI surgeries. Hepatic pseudoaneurysms have a high chance of ruptures (44%) with a high rate of mortality (as high as 50%) if not identified and managed promptly [9].

Since HAP may lead to sudden life-threatening hemorrhage, active treatment is required [10]. In cases of upper gastrointestinal (GI) bleeding where endoscopy cannot locate the source, CT angiography is used to identify rare causes like pseudoaneurysm of the right hepatic artery (RHA) with fistula formation in the GI and biliary tracts or an aorto-enteric fistula. Indicators of a ruptured pseudoaneurysm, aside from decreased hemoglobin, include worsening upper abdominal pain and jaundice due to hematoma compressing the bile duct. In our patient, both EUS and CT abdomen were inconclusive, but an MRI abdomen revealed a pseudoaneurysm of the RHA. Surgical treatment used to be the mainstream approach for managing visceral aneurysms, and due to advances in technology, embolization has become the preferred treatment for such lesions [2]. For patients at high risk of complications, such as liver failure or liver abscess, following hepatic artery embolization, implantation of covered stent grafts can be a viable alternative, as demonstrated in this case [10]. Patients with a history of gastrointestinal surgery resulting in hepatic artery or bile duct injury who present with gastrointestinal bleeding, shock, or unexplained abdominal pain should be evaluated for a possible pseudoaneurysm.
